# *In silico* Prediction of Protein–Protein Interaction Network Induced by Manganese II in *Meyerozyma guilliermondii*

**DOI:** 10.3389/fmicb.2020.00236

**Published:** 2020-02-19

**Authors:** France Anne Dias Ruas, Renata Guerra-Sá

**Affiliations:** Laboratório de Bioquímica e Biologia Molecular, Departamento de Ciências Biológicas, Instituto de Ciências Exatas e Biológica (NUPEB), Universidade Federal de Ouro Preto, Ouro Preto, Brazil

**Keywords:** *Meyerozyma guilliermondii*, manganese, bioremediation, proteome, protein–protein interactions, metabolic pathways

## Abstract

Recently, there has been an increasing interest in the use of yeast to produce biosorbent materials, because yeast is economical to use, adaptable to a variety of conditions, and amenable to morphological manipulations to yield better raw biomaterials. Previous studies from our laboratory have shown that *Meyerozyma guilliermondii*, a non-pathogenic haploid yeast (ascomycete), exhibits excellent biosorption capacity for Mn^2+^, as demonstrated by kinetic analyses. Shotgun/bottom-up analyses of soluble fractions revealed a total of 1257 identified molecules, with 117 proteins expressed in the absence of Mn^2+^ and 69 expressed only in the presence of Mn^2+^. In this article, we describe the first *in silico* prediction and screening of protein–protein interactions (PPIs) in *M. guilliermondii* using experimental data from shotgun/bottom-up analyses. We also present the categorization of biological processes (BPs), molecular functions (MFs), and metabolic pathways of 71 proteins upregulated in the *M. guilliermondii* proteome in response to stress caused by an excess of Mn^2+^ ions. Most of the annotated proteins were related to oxidation–reduction processes, metabolism, and response to oxidative stress. We identified seven functional enrichments and 42 metabolic pathways; most proteins belonged to pathways related to metabolic pathways (19 proteins) followed by the biosynthesis of secondary metabolites (10 proteins) in the presence of Mn^2+^. Using our data, it is possible to infer that defense mechanisms minimize the impact of Mn^2+^ via the expression of antioxidant proteins, thus allowing adjustment during the defense response. Previous studies have not considered protein interactions in this genus in a manner that permits comparisons. Consequently, the findings of the current study are innovative, highly relevant, and provide a description of interactive complexes and networks that yield insight into the cellular processes of *M. guilliermondii*. Collectively, our data will allow researchers to explore the biotechnological potential of *M. guilliermondii* in future bioremediation processes.

## Introduction

Heavy metal pollution represents one of the most serious global environmental problems. Although methods exist to minimize the environmental impact caused by these elements, traditional decontamination methods are known to release toxic products into the environment. The development of new ecological strategies for the efficient treatment of contaminated water is therefore a key target for researchers. Such strategies should be based on biological removal, or bioremediation, which is safer and more economically viable than traditional methods (Azubuike et al., 2016; Bahafid et al., 2017; Amorim et al., 2018).

Manganese (Mn) is a heavy metal that is soluble in water. Under normal circumstances, Mn is essential for growth, development, and homeostasis; however, serious health problems can arise when excessive amounts of Mn accumulate in the body (Erikson et al., 2005; Johnson and Hallberg, 2005; Zhao et al., 2010; Kafaei et al., 2017; Kornblith et al., 2018). In the state of Minas Gerais, Mn is naturally present in the soil and considered a natural constituent of the waters that drain from this region. However, Mn concentrations above the limits set out by environmental resolutions (Conselho Nacional do Meio Ambiente-Conama, 2005, 2011; Instituto Mineiro de Gestão das Águas [IGAM], 2012, 2014) have been found in this region. It is generally thought that this increase in Mn concentration is related to activities in the mining and metallurgical sectors. Indeed, Mn has been detected in the effluents and drains of almost all Brazilian mining sites (U.S. Department of the Interior and U.S. Geological Survey, 2012; Departamento Nacional de Produção Mineral [DNPM], 2017). A more significant increase in the levels of Mn was detected after the rupture of a dam in the city of Mariana-MG in 2015; dam residues were shown to contain large amounts of manganese. More recently, another dam broke, resulting in the further release of waste into the environment (Departamento Nacional de Produção Mineral [DNPM], 2017).

Traditionally, the removal of Mn is accomplished by the addition of strong oxidizing agents such as chlorine (Cl_2_), ozone (O_3_), and potassium permanganate (KMnO_4_); however, these agents generate toxic by-products. A potential alternative to these traditional processes is the use of biological technologies, such as bioremediation, that are more economically viable (Patil et al., 2016; Bahafid et al., 2017). The capacity of an organism for bioremediation, along with its tolerance or adaptability, is defined by molecular responses that are unique to each organism. Molecular techniques, such as proteomics and interactomics, allow us to identify and understand the metabolic potential of such organisms (Pandey and Mann, 2000; Mallick and Kuster, 2010). However, little is known about the molecular and biochemical mechanisms responsible for the accumulation and degradation of metals by yeast. In particular, the genetic and molecular basis of tolerance to heavy metals remains poorly understood. Consequently, there is an urgent need to identify the functional genes involved in tolerance and detoxification, and to elucidate the expression of key genes and proteins involved in the accumulation of heavy metals, along with their molecular interactions (Pieper and Reineke, 2000; Wase and Forster, 2003; Dixit et al., 2015; Bahafid et al., 2017).

Interactomics allows researchers to identify all types of molecular interactions, such as protein–protein interactions (PPIs), and examine metabolic networks that are regulated under certain physiological conditions. Analysis of PPIs has been employed in previous research to predict the function of uncharacterized proteins based on the fact that interacting proteins have similar functions (Feng et al., 2014; Cafarelli et al., 2017). Proteins participate in complex networks of biochemical interactions, at both functional and physical levels, between DNA, RNA, other proteins, lipids, and small metabolites. Developing an understanding of cellular mechanisms requires the analysis of interactomic networks using data acquired from proteomics. Oxidative stress caused by excessive levels of metals triggers an increase in gene expression, metabolic factors, and signaling proteins related to the stress response. By specifically analyzing PPIs, it is possible to identify all protein interactions that are upregulated or downregulated in certain environmental scenarios, thus providing clues for the identification of groups of molecules activated during the process of metal resistance; such research could be vital in developing biotechnological processes to facilitate the removal of Mn (Goll and Uetz, 2006; Instituto Mineiro de Gestão das Águas [IGAM], 2012; Feng et al., 2014; López et al., 2016; Patil et al., 2016; Cafarelli et al., 2017; Wang et al., 2018; Ruas et al., 2019).

*Meyerozyma guilliermondii* is a haploid, osmotolerant, non-pathogenic yeast that is able to utilize various carbon sources for survival and growth. Some strains exhibit physiological characteristics that can be used for the bioremediation of environmental metal contamination (Kaszycki et al., 2004; Butler et al., 2009; Kurtzman et al., 2011; Defosse et al., 2014). In a previous publication, we demonstrated that this strain of yeast may be employed in bioremediation processes to remove Mn^2+^ ions from water contaminated by mines. We confirmed this biosorptive removal capability using kinetic equations (Amorim et al., 2018), and recently described the first soluble proteome for this genus and this particular species (Ruas et al., 2019).

In the present study, we aimed to create the first PPI map for *M. guilliermondii* and describe the interaction network of proteins that are upregulated under stress conditions caused by an excess of Mn^2+^ ions. The present finding provides new insights into the underlying molecular and biochemical mechanisms, particularly the expression of upregulated proteins and the activation of metabolic pathways, under stress conditions caused by the excessive accumulation of Mn. We consider that the present findings related to the molecular mechanisms and kinetics of Mn removal will facilitate the development of new strategies for the application of this Mn^2+^-resistant yeast in biotechnological processes (Schwikowski et al., 2000; McDermott et al., 2005; Feng et al., 2014).

## Materials and Methods

This study involved a dataset that was previously published by our group (Ruas et al., 2019) and deposited in the ProteomeXchange Consortium (Vizcaíno et al., 2014) via the jPOST (Okuda et al., 2017) partner repository; this dataset can be accessed using the following dataset identifiers: <PXD010049> and <PXD PXD010050>. This dataset was obtained from *M. guilliermondii* yeast grown, for 7 days, in Yeast Peptone Dextrose (YPD) medium containing 2% (w/v) glucose, 1% (w/v) yeast extract, and 2% (w/v) peptone (pH 7.4) and under two different conditions: the absence and presence 0.91 mM of Mn^2+^ ions.

### LC-MS/MS Analysis

All experiments were conducted in biological duplicates and techniques triplicates. Proteins were extracted, dosed, and subsequently processed separately; 20 μg aliquots of protein fractions were separated by electrophoresis short runs in order to concentrate the proteins and eliminate contaminants of low molecular mass such as secondary metabolites. Analysis involved 500 ng of peptides obtained from the enzymatic digestion of biological replicates that were separated by gradient elution using an Acclaim PepMap100 C18 Nano-Trap column (75 μm id × 2 cm, 3 μm, 100 Å, Thermo Scientific) with an Acclaim PepMap100 C18 capillary column (75 μm id × 15 cm, 2 μm, 100 Å, Thermo Scientific). Ultrahigh performance liquid chromatography (UHPLC) separation of previously digested tryptic peptides was performed using the Dionex UltiMate^®^ 3000 UHPLC system (Thermo Scientific, Bremen). The spectra of biological duplicates were determined using a Q-Exactive^TM^ mass spectrometry instrument (Thermo Scientific). The instrument was operated at 1.9 kV in the positive mode with a resolution of 70,000 at 300–1750 *m/z*, a maximum injection time of 120 ms and a target value for automatic gain control (AGC) of 1 and 6 ions. We also used a 2 *m/z* window, fragmented by high energy collisional dissociation (HCD) with a normalized collision energy of 28–30 V. MS/MS spectra were obtained at a resolution of 17,500, a maximum injection time of 60 ms, and an AGC target value of 5 and 5 ions. After each MS/MS, we applied a subsequent dynamic deletion of 30 s (Ruas et al., 2019).

### Processing and Storage of Data in a Repository

Files containing raw data were subsequently searched by quantitative proteomics software package MaxQuant^®^, version 1.5.2.8 (Cox and Mann, 2008). Identified proteins were compared with prediction data in the *Meyerozyma guilliermondi* UniProt database, which contains 5,520 predicted sequences^1^ (accessed March 2017). In order to validate groups of proteins identified as being upregulated in the presence of an excess of Mn^2+^ ions in the biological duplicates, we used PEAKS software. We also used MaxQuant^®^ software to compare proteomic similarity and to determine unique proteins, as described previously (Kumar and Mann, 2009).

### Protein Interaction Datasets

Analyses were carried out *in silico* using a range of bioinformatics tools, as described below. Protein annotation was performed using UniProt Knowledgebase (UniProtKB) and proteins identified as “uncharacterized” were subjected to a Basic Local Alignment Search Tool (BLAST) search using the UniProtKB and BLASTp databases and considering a minimum sequence identity of 60%. Analyses of biological process (BP) and molecular function (MF) through Blast2GO Software^2^ via Gene Ontology (GO^3^) and KEGG (Ogata et al., 1999) (Kyoto Encyclopedia of Genes and Genomes^4^). The PSORT program was used to predict subcellular protein localization^5^, and biochemical pathways involving differentially expressed proteins were analyzed using TabPath^6^ (Moraes et al., 2017) and KEGG. Interaction analyses were performed using STRING^7^ (version 11.0), as described previously (Feng et al., 2014).

## Results

**FIGURE 1:**
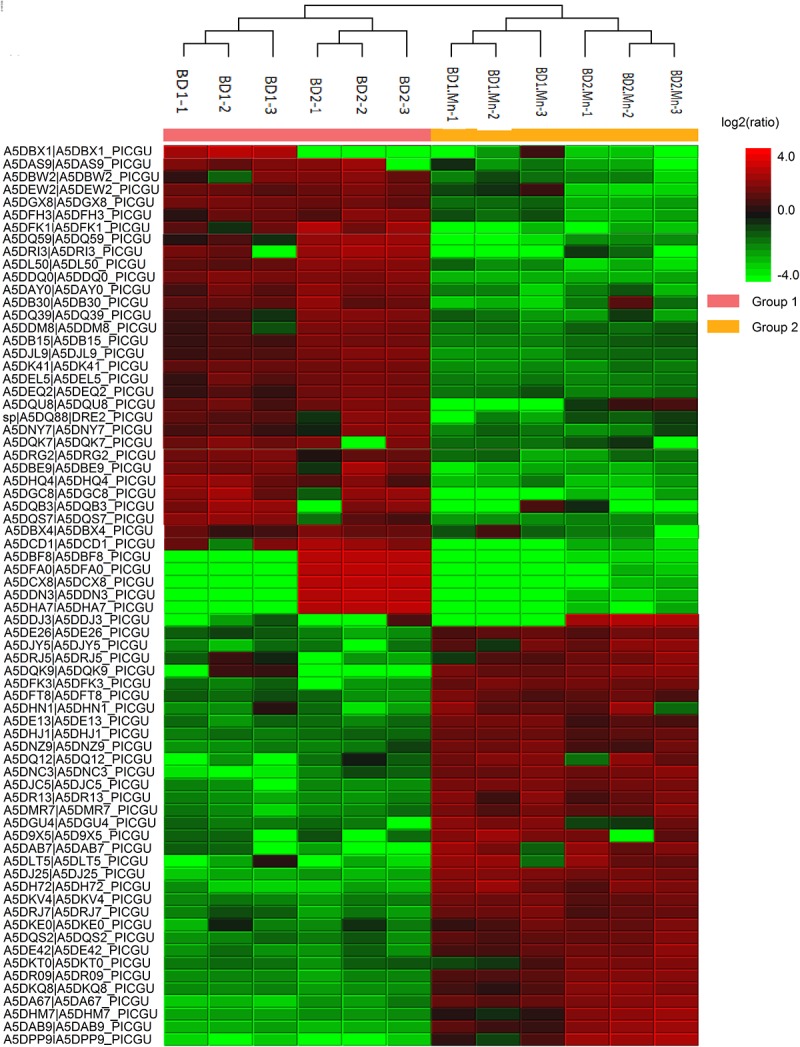
Heatmap representation of peptide quantification for the total proteome of *M. guilliermondii* based on the peak intensity of identified peptides from bottom-up proteomic data in the absence and presence of Mn^2+^.

**FIGURE 2:**
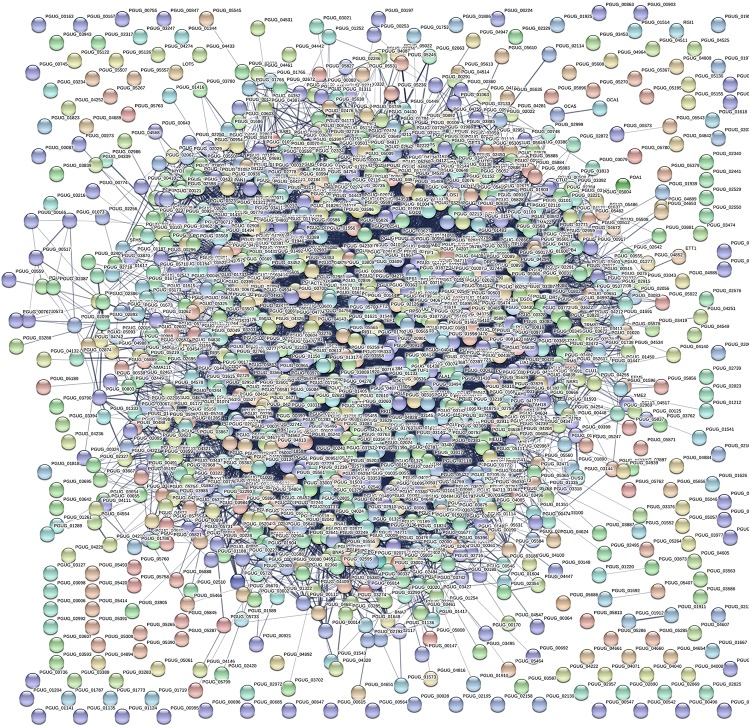
Network interactions of the 1257 proteins identified in the total soluble proteome of *Meyerozyma guilliermondii*. The most representative metabolic pathways were as follows: metabolic pathways (red), biosynthesis of secondary metabolites (blue), biosynthesis of antibiotics (green), biosynthesis of amino acids (yellow), carbon metabolism (lilac), glycolysis/gluconeogenesis cysteine and methionine metabolism (light blue), pyruvate metabolism (beige), methane metabolism (purple), and ribosome (brown) (PPI enrichment *p*-value: < 1.0 × 10^–16^).

We successfully identified 1257 proteins in the total proteome of *M. guilliermondii*. The heatmap shown in Figure 1 demonstrates that proteins were downregulated and upregulated under the two different physiological conditions (the absence and presence of Mn^2+^). Figure 2 and Supplementary Table S1 present for the first time the *in silico* prediction of the identified proteins, metabolic pathways, and the total proteome enrichment of the genus *Meyerozyma* sp. Results were obtained from the STRING database.

**FIGURE 3:**
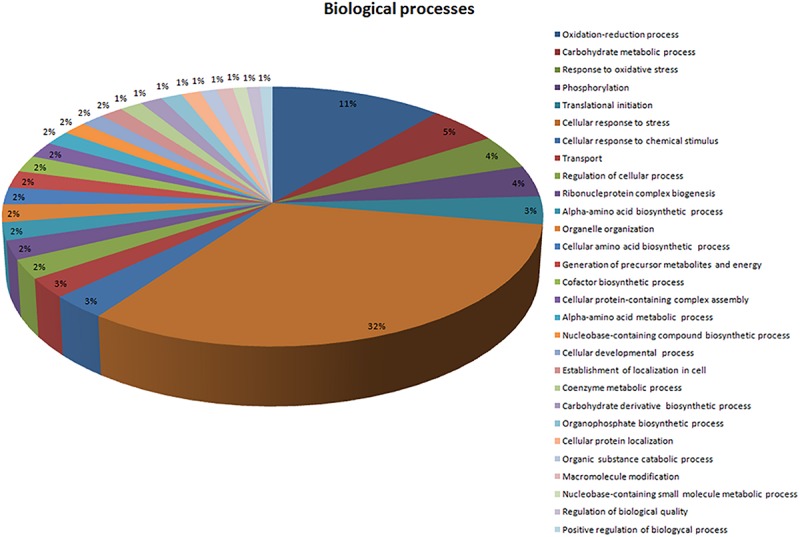
Description of the biological processes associated with upregulated proteins in the presence of Mn^2+^. Sequences were analyzed by Blast2GO and Gene Ontology (GO).

**FIGURE 4:**
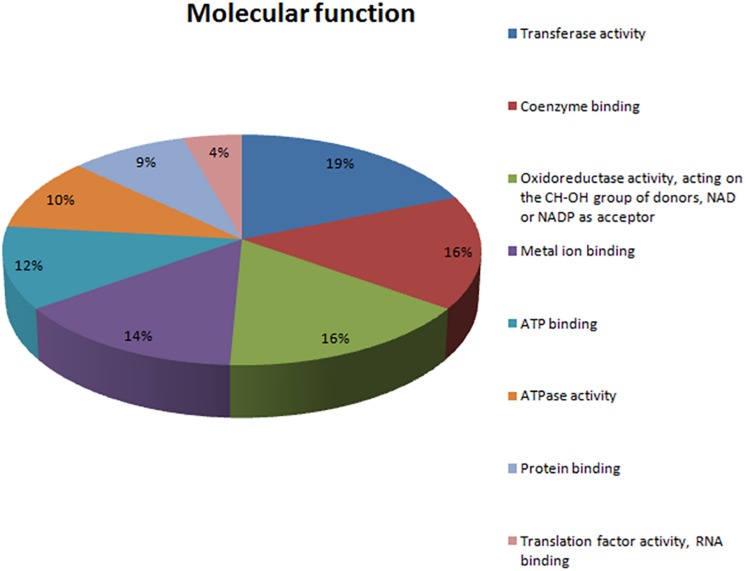
Categorization of the molecular functions of 71 upregulated proteins in the presence of excess Mn, as determined by the bioinformatic platforms Blast2GO and Gene Ontology (GO).

In total, 71 proteins were upregulated in the presence of an excess of Mn^2+^ ions. These positively regulated proteins in the presence of Mn^2+^ ions were classified according to the BPs in which they are involved (Figure 3) and their MFs (Figure 4). Most upregulated proteins relate to BPs associated with the cellular response to stress (32%), oxidation–reduction processes (11%), carbohydrate metabolic processes (5%), response to oxidative stress (4%), and phosphorylation (4%) (Figure 3). The most recurrent MFs in the differential proteome were transferase activity (19%), coenzyme binding (16%), oxidoreductase activity (16%), relative effect on the CH-OH group of donors (19%), NAD as an acceptor for the metal binding (14%), and ATP binding (12%) (Figure 4).

**FIGURE 5:**
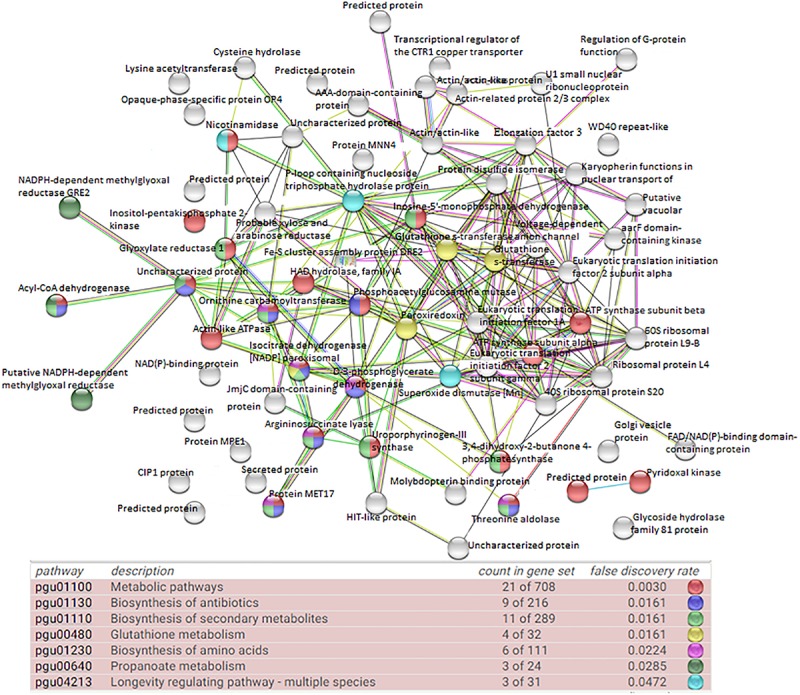
Network interactions of upregulated proteins in *M. guilliermondii* under excess Mn^2+^ stress, using tools and databases that can predict STRING protein function (PPI enrichment *p*-value: 5.29 × ^10–6*e*^).

The first PPI networks of *M. guilliermondii* total and differential proteome *in silico* were performed using the STRING, KEGG, and UniProt database (Figure 2), as well as the first demonstration of stress-rich functional enrichment analysis caused by Mn^2+^ (Figure 5). Seven functional enrichment analysis of the differentially caused by stress caused by Mn^2+^ were found: metabolic pathways, glutathione metabolism, biosynthesis of secondary metabolites, biosynthesis of antibiotics, biosynthesis of amino acids, propanoate metabolism, and longevity regulating pathway—multiple species (*p* = 5.29 × 10^–6^) (Figure 5 and Supplementary Table S2). Of these enrichments, the most representative pathway was metabolic pathways, followed by biosynthesis of secondary metabolites, antibiotics, and amino acids.

More specific analysis of the 71 upregulated proteins showed that they were associated with 42 metabolic pathways. The pathways associated with the largest number of proteins were metabolic pathways (19 proteins) followed by biosynthesis of secondary metabolites (10 proteins), biosynthesis of antibiotics (nine proteins), biosynthesis of amino acids (six proteins), glutathione metabolism (four proteins), and carbon metabolism (four proteins) (Table 2).

## Discussion

Previous studies from our group showed that *M. guilliermondii* possesses the ability to carry out manganese remediation via an efficient biosorption process. Due to these characteristics, this yeast can be effectively used for bioremediation of metal contamination in contaminated water. Results from the present study provide novel insights into molecular and biochemical mechanisms that allow this species of yeast to tolerate excessive concentrations of Mn^2+^ (Eccles, 1999; Shakya et al., 2015; Amorim et al., 2018).

In a previous study, we isolated *M. guilliermondii* and demonstrated that this species of yeast removes Mn^2+^ ions efficiently. Indeed, our experiments indicated that 100% of Mn^2+^ ions were removed from water in only 6 days (0.91 mM Mn^2+^); the removal kinetics were also indicative of efficient bioremediation (Amorim et al., 2018). Species that are capable of tolerating excessive concentrations of heavy metals may possess specific biochemical and molecular characteristics such as the expression of specific proteins and metabolic pathways that confer the ability to survive under heavy metal stress. We already know that proteins, their activities, and functions suffer interference from Mn^+2^, presented in the differential expression of proteins in Figure 1, and that the organism described herein uses such mechanisms to overcome stress conditions to the metal, thus generate a response that allows it to survive under conditions of excess metal. This yeast has mechanisms of gene alteration, differential protein expression, and activation of metabolic pathways that allow to correct and protect cellular and genetic integrity (Stohs and Bagchi, 1995; Kotrba and Ruml, 2000; Chen and Shi, 2002; Ercal et al., 2005; Wysocki and Tamás, 2010; Ruas et al., 2019).

In our previous paper, we reported that the majority of proteins in the proteome of *M. guilliermondii* are involved in BPs related to genetic and metabolic mechanisms (Ruas et al., 2019). The presence of excess Mn^2+^ ions caused stress to yeast, as expected, since most proteins that were upregulated in the presence of Mn^2+^ ions are mainly involved in BPs associated with cellular stress response (32%) (Figure 3). BPs associated with reduction and oxidation processes (11%) and oxidative stress response (4%) (Figure 3) also stood out. In general, oxidation–reduction processes act as catalysts for BPs that require electron transfer, such as metabolism and signaling processes that govern gene regulation and expression in certain situations (Prabhulkar et al., 2012). The proteins related to these processes are the majority in the differential proteome (Table 1).

**TABLE 1:** Differentially expressed proteins in the presence of Mn^2+^ ions.

**Acession**	**Gene**	**Description**	**e-Value**	**Sim mean**
A5DBF8	PGUG_00613	Voltage-dependent anion channel protein 2	0.0	92.15
A5DFA0	PGUG_01951	40S ribosomal protein S20	6,51E-73	94.34
A5DQK9	PGUG_05560	Uroporphyrinogen-III synthase	0.0	71.25
A5DCX8	PGUG_01133	ATP synthase subunit alpha, mitochondrial	0.0	96.25
A5DHA7	PGUG_02658	60S ribosomal protein L9-B	3,89E-130	94.64
A5DDN3	PGUG_01384	ATP synthase subunit beta, mitochondrial	0.0	97.92
A5DCD1	PGUG_00636	Actin-related protein 2/3 complex subunit 1	0.0	84.71
A5DGC8	PGUG_02329	Golgi vesicle protein	0.0	81.12
A5DFK1	PGUG_02052	Ornithine carbamoyltransferase	0.0	85.34
A5DQ59	PGUG_05410	Eukaryotic translation initiation factor 2 subunit gamma	0.0	94.51
A5DRI3	PGUG_05884	Karyopherin functions in nuclear transport of protein	2,02E-135	81.69
A5DDQ0	PGUG_01401	Eukaryotic translation initiation factor 1A	4,88E-70	98.69
A5DBE9	PGUG_00604	Glutathione S-transferase	2,27E-167	77.01
A5DBX1	PGUG_00776	Acyl-CoA dehydrogenase	0.0	87.75
A5DHQ4	PGUG_02805	Isocitrate dehydrogenase [NADP] peroxisomal	0.0	94.86
A5DQB3	PGUG_05464	NAD(P)-binding protein	0.0	77.64
A5DL50	PGUG_04001	Inosine-5’-monophosphate dehydrogenase	0.0	94.52
A5DAS9	PGUG_00384	Ribosomal protein L4	1,18E-169	78.33
A5DQS7	PGUG_05628	Argininosuccinate lyase	0.0	91.57
A5DAY0	PGUG_00435	Actin/actin-like protein	0.0	94.62
A5DGX8	PGUG_02529	Predicted protein	0.0	66.99
A5DK41	PGUG_03642	Opaque-phase-specific protein OP4	2,64E-156	72.65
A5DQU8	PGUG_05649	Putative vacuolar sorting protein	4,53E-160	77.31
A5DB30	PGUG_00485	Eukaryotic translation initiation factor 2 subunit alpha	0.0	93.07
A5DFH3	PGUG_02024	aarF domain-containing kinase	0.0	83.19
A5DBW2	PGUG_00767	Glycoside hydrolase family 81 protein	0.0	82.84
A5DJL9	PGUG_03470	P-loop containing nucleoside triphosphate hydrolase protein	0.0	92.9
A5DEW2	PGUG_01813	U1 small nuclear ribonucleoprotein	6,07E-129	67.83
A5DRG2	PGUG_056863	UDP-glucose 4-epimerase	0.0	81.85
A5DQ88	PGUG_05439	Fe-S cluster assembly protein DRE2	0.0	73.44
A5DEL5	PGUG_01716	D-3-phosphoglycerate dehydrogenase 1	0.0	94.77
A5DQ39	PGUG_05390	Secreted protein	0.0	68.28
A5DQK7	PGUG_05558	Pyridoxal kinase	0.0	73.51
A5DNY7	PGUG_04988	Regulation of G-protein function	0.0	73.48
A5DBX4	PGUG_00779	Actin/actin-like protein	0.0	97.87
A5DEQ2	PGUG_01753	Predicted protein	0.0	69.69
A5DB15	PGUG_00470	Elongation factor 3	0.0	95.71
A5DDM8	PGUG_01379	Protein MET17	0.0	91.27
A5DHJ1	PGUG_02742	3,4-Dihydroxy-2-butanone 4-phosphate synthase	4,37E-143	90.82
A5DKT0	PGUG_03881	WD40 repeat-like protein	8,77E-128	79.25
A5DFT8	PGUG_02139	Predicted protein	2,92E-112	80.51
A5DKE0	PGUG_03741	Glyoxylate reductase 1	0.0	83.28
A5DE26	PGUG_01527	Peroxiredoxin HYR1	1,91E-122	88.86
A5DE13	PGUG_01514	CIP1 protein	0.0	70.44
A5DNZ9	PGUG_05000	Phosphoacetylglucosamine mutase	0.0	81.25
A5DRJ5	PGUG_05896	Protein MNN4	0.0	74.75
A5DQS2	PGUG_05623	Nicotinamidase	3,75E-160	74.04
A5DRJ7	PGUG_05898	Probable xylose and arabinose reductase	0.0	78.81
A5DHN1	PGUG_02782	HIT-like protein	3,69E-125	80.15
A5DKV4	PGUG_03905	Cysteine hydrolase	1,02E-130	73.44
A5DE42	PGUG_01543	Lysine acetyltransferase (lysine N(6)-acetyltransferase) (LAT)	0.0	67.62
A5DGU4	PGUG_02495	FAD/NAD(P)-binding domain-containing protein	0.0	73.63
A5DMR7	PGUG_04568	Inositol-pentakisphosphate 2-kinase	0.0	78.13
A5D9X5	PGUG_00080	Threonine aldolase	0.0	86.86
A5DFK3	PGUG_02054	NADPH-dependent methylglyoxal reductase GRE2	1,76E-123	80.74
A5DR09	PGUG_05710	SEC14 cytosolic factor	0.0	89.81
A5DKQ8	PGUG_03859	Predicted protein	0.0	67.2
A5DR13	PGUG_05714	Putative NADPH-dependent methylglyoxal reductase GRP2	0.0	72.77
A5DQ12	PGUG_05363	Actin-like ATPase domain-containing protein	0.0	82.9
A5DJY5	PGUG_03586	Predicted protein	2,80E-110	65.64
A5DHM7	PGUG_02778	AAA-domain-containing protein	0.0	82.54
A5DJC5	PGUG_03376	JmjC domain-containing protein 4	0.0	69.22
A5DNC3	PGUG_04774	Protein disulfide isomerase	0.0	78.89
A5DDJ3	PGUG_01344	–		
A5DA67	PGUG_00172	Superoxide dismutase [Mn], mitochondrial	4,53E-148	81.76
A5DJ25	PGUG_03276	Glutathione S-transferase	2,58E-170	76.38
A5DAB9	PGUG_00224	Transcriptional regulator of the CTR1 copper transporter	0.0	62.06
A5DLT5	PGUG_04236	Predicted protein	0.0	60.57
A5DAB7	PGUG_00222	Molybdopterin binding protein	0.0	78.3
A5DH72	PGUG_02623	HAD hydrolase, family IA	0.0	92.99
A5DPP9	PGUG_05250	Protein MPE1	3,27E-159	80.88

The key MFs of this expressed proteome are protein binding, transferase activity, and oxidoreductase activity (Figure 4). These results corroborate the number of BPs and demonstrate the capacity of the studied species under stressful conditions triggered by Mn^2+^. It has become clear that this species expresses proteins that activate detoxification regulatory mechanisms which allowed it to tolerate considered Mn^2+^ concentrations (Hohmann and Mager, 2003; Wysocki and Tamás, 2010; Ruas et al., 2019).

Stressful conditions in yeast triggered by the presence of Mn^2+^ upregulated the expression of 71 proteins. Some of the upregulated proteins were directly related to protein biosynthesis, thus confirming the need for the synthesis of protective proteins in response to overexposure to the tested ions. Others, most of them, are related to oxidation–reduction, metabolism, and oxidative stress response processes. These results confirm that *M. guilliermondii* has molecular and biochemical mechanisms to overcome Mn^2+^ toxicity. The effects of metal toxicity on metabolism and homeostasis in *M. guilliermondii*, particularly in response to Mn exposure, are not known, therefore, the need for this report.

We know that the exposure of cells to excessive concentrations of heavy metals causes an increase in the cellular levels of reactive oxygen species (ROS) that impair and interfere with various cellular components, such as nucleic acids and proteins in yeasts. Heavy metals can cause damage in yeast cells via increased levels of ROS, which lead to the abnormal regulation of redox-sensitive pathways, and through oxidative changes to essential biomolecules (Fridovich, 1998; Shanmuganathan et al., 2004; Ercal et al., 2005; Schrader and Fahimi, 2006; Bonekamp et al., 2009; Pan et al., 2017).

Thus, *M. guilliermondii* activates these oxidative control mechanisms. Most proteins found in the proteome are related to protein oxidoreductase activity, oxidative stress response, and mitochondrial activity and peroxisomes. This reduction activity is confirmed by the protein interaction network in Figure 5 and the pathway enrichment functional analysis (Table 2). We found upregulated acyl-CoA dehydrogenase (A5DBX1) that catalyzes the early stage of the mitochondrial fatty acid β-oxidation cycle (Colin and Kimt, 1995). Isocitrate dehydrogenase [NADP] peroxisomal (A5DHQ4), which acts on the krebs cycle by providing NADPH for maintenance of the reduced glutathione and peroxiroxin systems (Table 1 and Supplementary Table S2) (Smolková and Ježek, 2012). The Peroxiredoxin (Prx) (A5DE26) is involved in redox homeostasis and oxidative stress (Bonekamp et al., 2009; Murray et al., 2011; Nelson et al., 2011; Soito et al., 2011) and glutathione S-transferase (A5DBE9, A5DJ25) plays essential role in antioxidant activity (Stohs and Bagchi, 1995).

**TABLE 2:** Metabolic pathways of proteins related to manganese tolerance in differential proteomes.

**Pathway**			**Accession**	
Metabolic pathways	A5DFK1 A5DDM8 A5DBX1 A5DCX8 A5DQK7	A5DQS7 A5DEL5 A5DQK9 A5DDN3 A5DQ12	A5DHQ4 A5DRG2 A5DHJ1 A5DMR7 A5DH72	A5D9X5 A5DL50 A5DQS2 A5DNZ9
Biosynthesis of secondary metabolites	A5DFK1 A5DDM8 A5DQK9	A5DQS7 A5DRG2 A5DHJ1	A5DHQ4 A5DL50	A5D9X5 A5DBX1
Biosynthesis of antibiotics	A5DFK1 A5DDM8 A5DNZ9	A5DQS7 A5DEL5	A5DHQ4 A5DRG2	A5D9X5 A5DBX1
Biosynthesis of amino acids	A5DFK1 A5DDM8	A5DQS7 A5DEL5	A5DHQ4	A5D9X5
Glutathione metabolism	A5DHQ4	A5DE26	A5DJ25	A5DBE9
Carbon metabolism	A5DHQ4	A5DDM8	A5DEL5	A5DBX1
Propanoate metabolism	A5DBX1	A5DFK3	A5DR13	
Ribosome	A5DFA0	A5DAS9	A5DHA7	
RNA transport	A5DQ59	A5DB30	A5DDQ0	
Longevity regulating pathway	A5DQS2	A5DJL9	A5DA67	
Oxidative phosphorylation	A5DCX8	A5DDN3		
Arginine biosynthesis	A5DFK1	A5DQS7		
Purine metabolism	A5DL50	A5DHN1		
Glycine, serine, and threonine metabolism	A5D9X5	A5DEL5		
Amino sugar and nucleotide sugar metabolism	A5DRG2	A5DNZ9		
Pyruvate metabolism	A5DFK3	A5DR13		
MAPK signaling pathway	A5DFK3	A5DR13		
Protein processing in endoplasmic reticulum	A5DNC3	A5DB30		
Peroxisome	A5DHQ4	A5DA67		
Glycolysis/gluconeogenesis	A5DRG2			
Citrate cycle (TCA cycle)	A5DHQ4			
Pentose and glucuronate interconversions	A5DQ12			
Galactose metabolism	A5DRG2			
Fatty acid degradation	A5DBX1			
Alanine, aspartate, and glutamate metabolism	A5DQS7			
Cysteine and methionine metabolism	A5DDM8			
Valine, leucine, and isoleucine degradation	A5DBX1			
Beta-alanine metabolism	A5DBX1			
Glycerolipid metabolism	A5DH72			
Inositol phosphate metabolism	A5DMR7			
Arachidonic acid metabolism	A5DE26			
Methane metabolism	A5DEL5			
Riboflavin metabolism	A5DHJ1			
Vitamin B6 metabolism	A5DQK7			
Nicotinate and nicotinamide metabolism	A5DQS2			
Porphyrin and chlorophyll metabolism	A5DQK9			
Sulfur metabolism	A5DDM8			
2-Oxocarboxylic acid metabolism	A5DHQ4			
Fatty acid metabolism	A5DBX1			
mRNA surveillance pathway	A5DPP9			
Phosphatidylinositol signaling system	A5DMR7			
Endocytosis	A5DCD1			

In studies with *Saccharomyces cerevisiae*, it was observed that metals and metalloids, such as cadmium (Cd), arsenic III (As III), among other metals, were neutralized. Neutralization triggers a mechanism of glutathione depletion (GSH), usually associated with metal toxicity. GSH depletion influenced the redox environment and impaired the activities of GSH dependent enzymes such as glutathione peroxidases, glutathione S-transferases, and glutaredoxins, affecting many cellular processes (Stohs and Bagchi, 1995). *M. guilliermondii* demonstrated that GSH depletion probably does not occur due to the presence of Mn^2+^. Two glutathione S-transferase (A5DBE9, A5DJ25) have been identified, confirming with data that this yeast has very efficient mechanisms to overcome the stress caused by Mn^2+^ by protein expression and activation of antioxidant pathways in its cells.

Another protein with important oxidative reduction activity has been expressed as superoxide dismutase [Mn] mitochondrial (A5DA67), well known as a metalloenzyme that acts against oxidative stress and plays a key role in cellular defense (Ribeiro et al., 2017). We believe that *M. guilliermondii* attenuate damage caused by metals and ROS via the expression of proteins and antioxidant systems, thus allowing ecotoxicological defense response (Fridovich, 1998; Hohmann and Mager, 2003; Hosiner et al., 2014).

We identified using TabPath metabolic pathways of glycolysis/gluconeogenesis (UDP-glucose 4-epimerase—A5DRG2) e via peroxisome (isocitrate dehydrogenase [NADP] peroxisomal—A5DHQ4; superoxide dismutase—A5DA67) (Table 2). Previous research has shown that heavy metal toxicity increases the activity of enzymes such as glucose-6-phosphate dehydrogenase and peroxidases. Heavy metals can also affect plasma membrane lipids by modifying membrane properties linked to permeability, fluidity, the modulation of membrane-bound ATPase activities, and interfere with proper protein folding (Niki, 2009; Jan et al., 2015).

ATPase activity was identified as one of the MFs that was significantly represented in the proteome of *M. guilliermondii* (10%) (Figure 4). Damage to cell membrane ATPase activity and increased lipid peroxidation are indication of increased oxidative stress caused by metals (Niki, 2009). Adaptive response to lipid peroxidation requires the transcriptional regulation of antioxidant genes, including those encoding enzymes related to glutathione synthesis and metabolism (four proteins: A5DHQ4, A5D26, A5DJ25, A5DBE9) and two glutathione S-transferases (A5DJ25 and A5DBE9) that play a key role in redox homeostasis, cell signaling, and detoxification (Figure 5 and Table 2) (Niki, 2009; Zhang et al., 2013; Jan et al., 2015; Özaslan et al., 2017). The functional enrichment analysis results show the glutathione metabolism pathway as one of the highlights, just as PPI networks show four proteins related to the same functions including two glutathione S-transferase (A5DBE9 and A5DJ25), peroxiredoxin (A5DE26), and peroxisomal isocitrate dehydrogenase [NADP] (A5DHQ4).

Certain metabolic pathways are activated allowing stress to be overcome by detoxification or metal elimination mechanisms. Some induce the precipitation of heavy metals, including metal carbonates, phosphates, and sulfates. Other pathways allow stress to be overcome by mechanisms of detoxification and the elimination of metals. Species that have the ability to adapt or tolerate metals are of very important and could potentially be utilized in important biotechnological processes (Kotrba and Ruml, 2000; Ercal et al., 2005).

*Meyerozyma guilliermondii* upregulated proteins are involved in 42 metabolic pathways, according to TabPath analysis. Functional enrichment analysis demonstrated seven-way enrichment and that proteins have a network of interactions between proteins that confirm the results of analyzes of biological functions and processes. This pathway relates to several metabolic reactions (catabolism and anabolism) that are necessary for carrying out all processes for body maintenance, including glycolysis, pentose pathway, electron transported chain, and others. Ten identified proteins belong to biosynthesis of secondary metabolites, which in yeast involves many proteins and protein complexes that allow it to respond to various environmental stimuli such as stress to metals, just as *M. guilliermondii* was exposed (Fox and Howlett, 2008). Other pathways that have been observed as defense reactions and detoxification mechanisms in yeast due to oxidative damage induced by heavy metals were biosynthesis of amino acids, propanoate, and longevity regulating pathway (Figure 5 and Supplementary Table S2).

Species that possess the ability to remove heavy metals may exhibit adaptive characteristics such as specific metabolic pathways for the remediation of metals. Certain metabolic pathways mediate the precipitation of heavy metals into carbonates, phosphates, and metal sulfates with potential biotechnological applications (Kotrba and Ruml, 2000). In addition, some species can produce metal-binding compounds, such as bio-pigments (flavins, flavonoids, polyhydroxy anthraquinones, and tannin), which contribute to the stress response induced by exposure to metals by acting as the first barrier against a variety of metals. These binders are predominant in metal-binding sites, when dead biomass is used as a biosorbent material (Kotrba and Ruml, 2000; Farooq et al., 2010). Indeed, a recent study involving *M. guilliermondii* reported that dead biomass removed manganese ions more efficiently than live biomass (Amorim et al., 2018), suggesting that metal binding compounds may play a role in this process.

In addition to producing metal binding compounds, biosynthesis of secondary metabolites, amino acids, antibiotics, activation of metabolic pathways also contribute to success in a such stressful situation. Consequently, these factors are of vital importance for the decontamination of metals and thus present meaning for possible biotechnological applications (Holan and Volesky, 1995; Kotrba and Ruml, 2000; Ahemad and Kibret, 2001; Farooq et al., 2010). The possibility of altering the properties of living species used in heavy metal remediation and construction of chimeric organisms that have desirable characteristics using genetic engineering are now under study in many laboratories.

## Conclusion

Heavy metal removal processes by bioremediation represent an economical alternative to physicochemical decontamination methods. In this paper, we describe the molecular interactions of a yeast strain, *M. guilliermondii*, with proven resistance to Mn^2+^ ions. Collectively, our findings allow us to identify the molecular interactions associated with proteins that are upregulated in the presence of Mn^2+^ and describe the metabolic pathways that play a role in the expression of these proteins. The majority of the upregulated proteins were related to oxidoreductase activity. Our findings indicate that *M. guilliermondii* is able to tolerate excessive Mn^2+^ concentrations via the expression of antioxidant proteins, thus creating a more effective ecotoxicological defense response. Our data corroborate the findings of previous studies in that this species has significant potential for application in biotechnological processes used to remove Mn^2+^ contamination from water. The results presented here, in addition to contributing to scientific knowledge about the species, may help to develop future methods for monitoring potentially hazardous and toxic manganese-contaminated areas or waters (rivers, dams). It may further contribute to the establishment of future standardized tests using the species *M. guilliermondii* or even employ as a suitable eukaryotic organism model for bioremediation processes.

## Data Availability Statement

Our mass spectrometry proteomics data have been deposited in the ProteomeXchange Consortium [49] via the jPOST [50] partner repository with the following dataset identifiers: <PXD010049> and <PXD010050>.

## Author Contributions

FR designed and performed Mn^2+^ experiments with *M. guilliermondii*, processed the data, carried out bioinformatic analyses and statistical analysis, deposited the proteomic dataset in public repositories, and wrote and reviewed the manuscript. RG-S designed and supervised the study and reviewed the manuscript. Both authors read and approved the final manuscript.

## Conflict of Interest

The authors declare that the research was conducted in the absence of any commercial or financial relationships that could be construed as a potential conflict of interest.

## Footnotes

1 http://www.uniprot.org/uniprot/?query=Meyerozyma+guilliermondii&sort=score

2 https://www.blast2go.com/

3 http://www.geneontology.org/

4 https://www.genome.jp/kegg/pathway.html

5 https://www.psort.org/psortb/

6 http://200.239.132.160:8686/

7 https://string-db.org/
